# Analysis of the Health Product Profile Directory — a new tool to inform priority-setting in global public health

**DOI:** 10.1186/s12961-019-0507-1

**Published:** 2019-12-12

**Authors:** R. F. Terry, A. Plasència, J. C. Reeder

**Affiliations:** 1 TDR, the Special Programme for Research and Training in Tropical Diseases, Geneva, Switzerland; 2 ISGlobal, Barcelona Institute for Global Health, Barcelona, Spain; 3 Hospital Clínic-Universitat de Barcelona, Barcelona, Spain

**Keywords:** Health Product Profile Directory, R&D, TDR, WHO

## Abstract

**Background:**

The Health Product Profile Directory (HPPD) is an online database describing 8–10 key characteristics (such as target population, measures of efficacy and dosage) of product profiles for medicines, vaccines, diagnostics and other products that are intended to be accessed by populations in low- and middle-income countries. The HPPD was developed by TDR on behalf of WHO and launched on 15 May 2019.

**Methods:**

The contents of the HPPD were downloaded into an Excel™ spreadsheet via the open access interface and analysed to identify the number of health product profiles by type, disease, year of publication, status, author organization and safety information.

**Results:**

The HPPD contains summaries of 215 health product profiles published between 2008 and May 2019, 117 (54%) of which provide a hyperlink to the detailed publication from which the summary was extracted, and the remaining 98 provide an email contact for further information. A total of 55 target disease or health conditions are covered, with 210 profiles describing a product with an infectious disease as the target. Only 5 product profiles in the HPPD describe a product for a non-communicable disease. Four diseases account for 40% of product profiles in the HPPD; these are tuberculosis (33 profiles, 15%), malaria (31 profiles, 14%), HIV (13 profiles, 6%) and Chagas (10 profiles, 5%).

**Conclusion:**

The HPPD provides a new tool to inform priority-setting in global health — it includes all product profiles authored by WHO (*n* = 51). There is a need to standardise nomenclature to more clearly distinguish between strategic publications (describing research and development (R&D) priorities or preferred characteristics) compared to target product profiles to guide a specific candidate product undergoing R&D. It is recommended that all profiles published in the HPPD define more clearly what affordability means in the context where the product is intended to be used and all profiles should include a statement of safety. Combining the analysis from HPPD to a mapping of funds available for R&D and those products in the R&D pipeline would create a better overview of global health priorities and how they are supported. Such analysis and increased transparency should take us a step closer to measuring and improving coordination of efforts in global health R&D.

## Background

In this paper, we analyse the content of the new Health Product Profile Directory (HPPD) developed by TDR (the Special Programme for Research and Training in Tropical Diseases) and assess the potential of HPPD as a new tool to inform product priority-setting for global health. The HPPD is a searchable database describing 8–10 key characteristics (such as target population, measures of efficacy and dosage) of profiles for medicines, vaccines, diagnostics and other products. The HPPD has specific criteria to include only those product profiles that describe health products intended to be accessed by populations in low- and middle-income countries where conventional research and development (R&D) market incentives do not function for diseases sometimes described as neglected diseases. The HPPD is a global public good created by WHO as part of its efforts to improve access to medicines and achieve universal health coverage.

A product profile is a tool to plan and incentivise R&D and can be used to frame the description of a product for regulatory purposes [1]. The product profile in different forms can serve different purposes. The Target Product Profile (TPP) provides strategic oversight to optimise the development of existing product candidates within the R&D pipeline. TPPs have primarily been used by regulatory bodies and the pharmaceutical industry since approximately 2005 [2], although we now see this term also used in the not-for-profit space, for example, by the Product Development Partnerships (PDP) [3].

Nevertheless, product profiles can also be written as strategic documents describing an ideal or preferred set of characteristics for a product that does not exist as a way of setting a priority and guiding innovation in that area. For WHO, these strategic profiles, often termed Preferred Product Characteristics (PPC), aim to provide a technical description of the characteristics (for example, target population, dosage, efficacy) that the new product will need to have to tackle that health priorities effectively. In keeping with its mandate, WHO preferences reflect its desire to promote the development of new products with high public health impact that are suitable for use in low- and middle-income countries.

Using vaccine development as an example, a WHO PPC document describes the indications, target groups, possible immunisation strategies and features of the desired clinical data related to safety and efficacy. These preferences are related to the unmet public health need in a priority disease area for which WHO encourages vaccine development. A recent example includes vaccine development for Ebola, where the commercial return on a new product may not be sufficient to support the necessary R&D but the potential risk to public health is great. The PPC does not describe minimally acceptable criteria. Each PPC addresses early stage vaccine R&D, generally at least 5–10 years from vaccine availability, and they are reviewed and updated if necessary at least every 5 years. PPC are not static exit criteria but are structured in such a way so as to drive innovation for early-stage products to address public health needs [4].

The issue of coordinating and financing R&D for products where markets are failing was the subject of a TDR report in 2016. Following a request from the member states of WHO, TDR undertook research that described how a new global fund supporting R&D for neglected diseases could be developed and managed. The subsequent TDR report, Health Product Research and Development Fund: a Proposal for Financing and Operation, developed a new R&D modelling tool to predict the impact of seven funding scenarios, ranging from an annual disbursement of $1 million to $500 million, to improve global health R&D funding and coordination [5, 6].

The 2016 report also highlighted the need for a global mechanism to identify and communicate R&D priorities and coordinate efforts to meet these. In the consultations undertaken for that report, product profiles were identified as a tool that was becoming more widely used by WHO, industry, the PDPs and funding agencies to manage their R&D portfolios. The report concluded that, if these product profiles could be summarised in a standard template and brought together in a database directory, this would improve access to information at a global level on health product R&D. The more precise description of public health needs contained in a product profile would strengthen the efforts to develop new health products where conventional markets were absent, for example, the poverty-related neglected diseases, emerging pandemics or novel antibiotics. In addition, a directory would enable a high-level analysis of global health priorities, identify where there are gaps, and inform a discussion on what norms and standards are need for subsequent product profile development.

Subsequently, TDR undertook a series of further consultations with WHO staff and external stakeholders engaged in global health product R&D (as listed in Annex 2 of the 2016 TDR Report [5]) to develop this online resource. Stakeholders were requested to provide a link to a published product profile or to provide the meta-data if there was no public document. The information was collated and transposed to a standard template summary or meta-data captured in a HPPD record (Table 1).

**Table 1:** Description of the health product profile characteristics contained in the Health Product Profile Directory (please note, not all characteristics apply to all profiles)

Profile characteristic	Example descriptions
Document title	A description of the health product profile, including product type, target disease and/or health condition for a target population or geographic location
Author	The lead organisation that developed and published the health profile. When a profile was published in a journal, the main institutional affiliation of the corresponding author was chosen
WHO	A check-box to enable quick separation of WHO-authored health product profiles from those authored by others
Diseases	One or more diseases and/or health conditions (e.g. reproductive health) targeted by the product
Product type	Short-description of the product type: diagnostic, drug, vaccine, digital health, drug regimen, injectable/implant, not defined. As the directory content grows, these definitions will expand
Year published	The year the profile document was published
Status	Active: the profile is still considered relevant Archive: the profile is no longer relevant. In the Health Product Profile Directory, profiles older than 5 years from the current date are considered archive
Indication	The purpose of the product, e.g. to provide immunisation against a given disease or to identify the presence of a given bacteria in drinking water
Intended use	Primarily used for diagnostics to separate triage, screening and more precise diagnosis
Target population	Age group or other specific subpopulation groups
Sample type and volume	Primarily for diagnostics describing the medium that is tested, e.g. blood, stool, saliva, drinking water
Use setting	Who would use the product and under what conditions, infrastructure requirements, e.g. trained nurse in a low-resource setting, primary healthcare facility, no cold chain, shelf life
Performance	Describes for diagnostics: specificity, sensitivity, reproducibility, robustness, time to result, nature of result, qualitative or quantitative
Efficacy	For drugs and drug regimens: the clinical characteristics, dosing, pharmaco-dynamics, rate of onset of action, interaction with other therapeutics, etc. For vaccines: expected efficacy, duration, reversibility, strain coverage, interaction with other vaccines, etc.
Safety	For drugs, drug regimens and vaccines: clinical safety and tolerability, safety monitoring requirements, contra-indications and relation to specific population types, e.g. infants, pregnant women, during breast-feeding
Comments	Additional comments not able to be categorized above
Document url	Ideally, an archive providing a permanent url where the full document is published openly on line
Contact email	A contact email for further information

The criteria for inclusion of a product profile in the HPPD are (1) product profiles with a focus on addressing health issues in low- and middle-income countries for which there is no market or limited incentive for R&D; (2) health product profiles covering pharmaceuticals/therapeutics, biologics, vaccines, diagnostics, medical devices/equipment; (3) all product profiles prioritized for global action by WHO and its technical departments (these were marked as authored by WHO); and (4) all product profiles authored by other UN agencies, PDPs, commercial companies and other organisations that share the same inclusion criteria listed above (these are marked as non-WHO profiles).

The first publication of HPPD was by design not intended to provide a comprehensive analysis of all health products in the pipeline.

The data were collated in a Microsoft Excel™ spreadsheet used to populate a web-based database on the TDR website using Sitefinity™ software. The HPPD was launched on 15 May 2019 [7, 8]. This database includes a form to submit a new product profile online given that one of the key aims of HPPD is to provide up-to-date information. Users are strongly encouraged to read the Frequently Asked Questions document on this site before using the HPPD or submitting a new profile [9].

## Methods

The HPPD was accessed on 31 May 2019. An output of the whole content of the HPPD was generated by using the search function with no specific search values selected. The HPPD creates a .CSV file, which was downloaded and saved into Microsoft Excel™ 2016. The Excel file was then formatted and filters were added to enable an analysis of the number of health products by type, disease, year of publication, status, author organization and safety information. Twenty profiles published between 2008 and 2013 were given an ‘Archive’ status as internal best practice at WHO recommends that profiles older than 5 years should be updated if they are to be relevant in guiding R&D. The analysis presented here includes all 215 records comprising both Archive and Active profiles.

## Results

On 31 May 2019, the HPPD contained summaries of 215 health product profiles dating from 2008 through to May 2019. Of these, 117 (54%) provide a hyperlink to the detailed publication from which the summary was extracted, with the remaining 98 providing an email contact for further information. Of the 117 with a published document, 63 published the profile on the website of the authoring organization, 31 were published in an online academic journal and 23 were published in the digital repository of WHO, called IRIS, with a link to the relevant WHO webpage [10]. Publishing a product profile in a journal or a digital repository should be encouraged as it provides an archive-quality, permanent url reference for the publication. Publications on websites are at risk of the link breaking when webpages are updated and are unreliable as references for documents of this nature.

All the profiles could be categorised as strategic documents in that they describe an ideal or preferred set of characteristics. None of the documents provide any link to a specific candidate that is undergoing R&D. There is a wide variation of terms used to describe the profiles with the term ‘Target Product Profile’, or a variation of that phrase, used in 52 of the profiles. The HPPD contains links to 10 WHO profiles for vaccines that are entitled ‘Preferred Product Characteristics’ and the Bill and Melinda Gates Foundation has 12 profiles that describe an intervention as Target Product Profile. Both the PPC and intervention TPPs are more explicit in describing their purpose as strategic. However, due to the absence of standard nomenclature, it is not reliable to infer the purpose of a document in the HPPD from the title. Similarly, it should be noted that, in the analysis presented here, each record was given equal weight and no assessment to distinguish between the profiles with respect to quality was made. For example, of the 129 profiles that describe a drug or vaccine, 58 (45%) of these contain no reference to safety requirements. As the HPPD is the first resource of its kind, the increased visibility and ease of comparing profiles across categories should act as a driver to improve standards and quality over time.

In addition to the typical health product profiles for diagnostics, drugs, vaccines and drug regimens, the HPPD also contains other product profiles, including seven profiles describing software to manage tuberculosis (TB) patient data, products for injectable or implant contraception, and other profiles describing contraceptive methods for a target population where the specific product type was undefined.

**Fig. 1:**
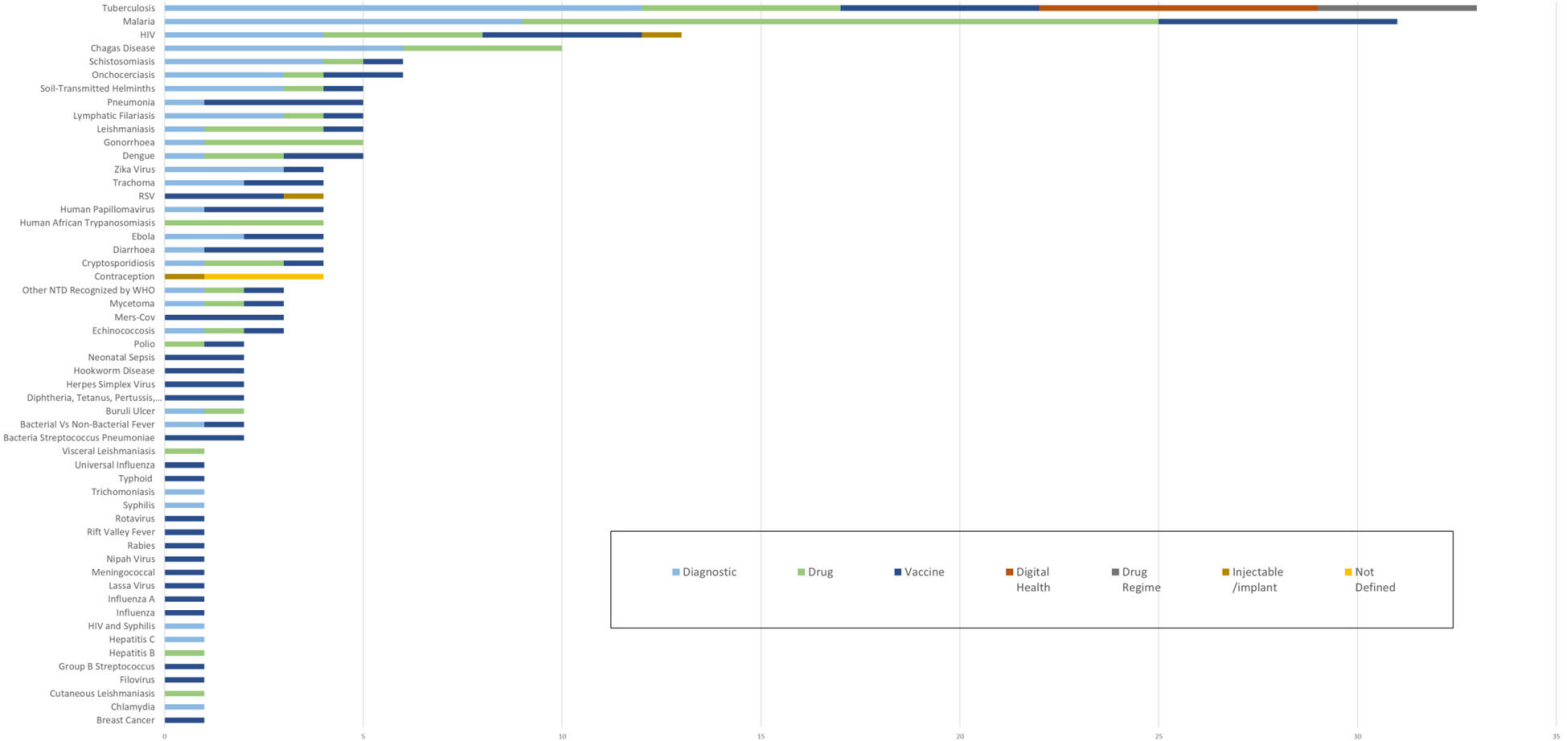
Number of health product profiles by disease (May 2019, *n* = 215)

Fifty-five target disease or health conditions were covered by the 215 health product profiles, with 210 profiles describing a product with an infectious disease as the target (Fig. 1). Only five product profiles or less than 2% of the profiles in the HPPD describe a product for a non-communicable disease (NCD) (one vaccine for breast cancer and four contraception technologies). The top four diseases accounting for 40% of product profiles in the HPPD were TB, malaria, HIV and Chagas (Table 2).

**Table 2:** The four diseases with the highest number of product profiles in the Health Product Profile Directory (May 2019)

Tuberculosis	33 profiles (15%)	Diagnostic (12), Drug (5), Vaccine (5), Digital Health (7), Drug Regimen (4)
Malaria	31 profiles (14%)	Diagnostic (9), Drug (16), Vaccine (6)
HIV	13 profiles (6%)	Diagnostic (4), Drug (4), Vaccine (4), Injectable/implant (1)
Chagas	10 profiles (5%)	Diagnostic (6), Drug (4)

All of the emerging diseases with potential to cause pandemics identified by WHO in the R&D Blueprint are covered by one to three product profiles describing diagnostics, drugs and vaccines [11]. By comparison, many of the neglected tropical diseases have limited representation in the profiles, with many only having one or two profiles.

**Fig. 2:**
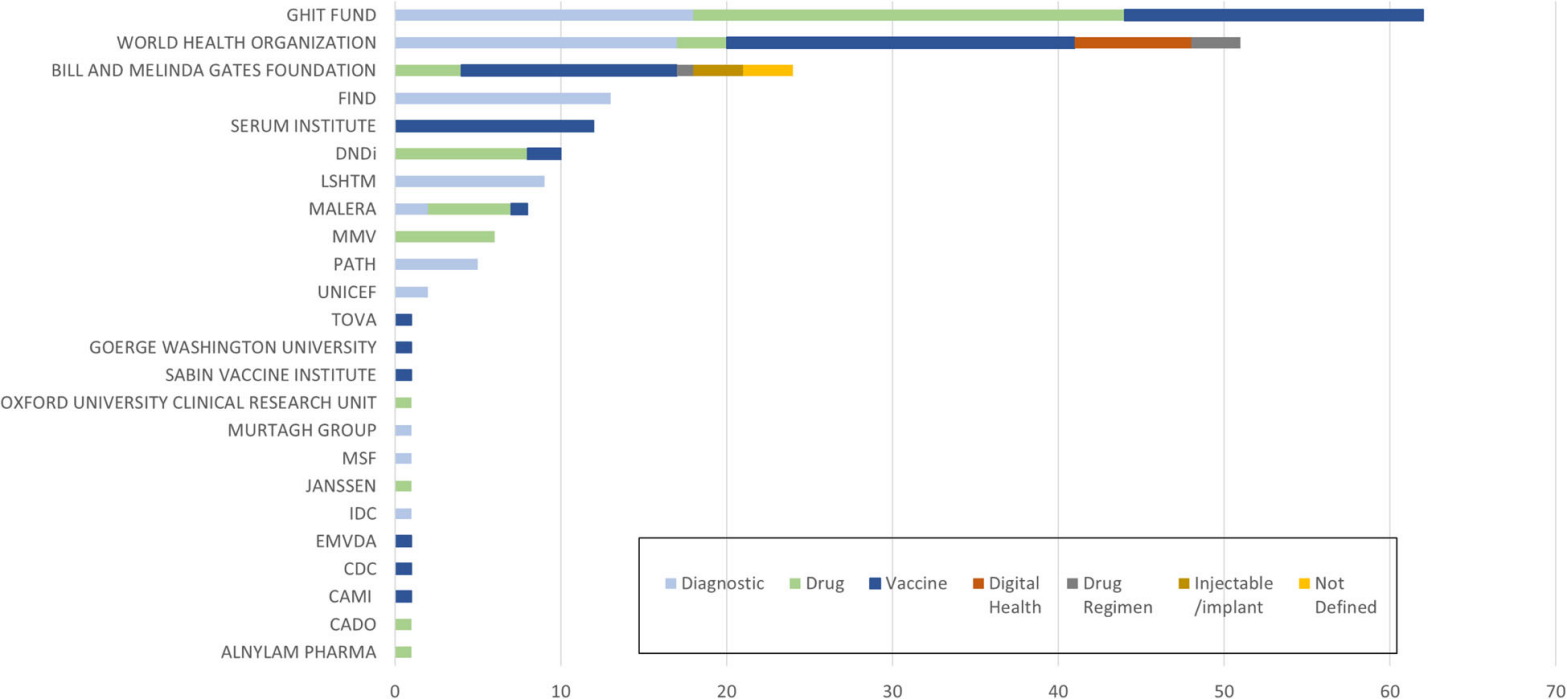
Product profiles by organisation author (May 2019, *n* = 215)

Of the 215 profiles, 16 (7%) are authored by for-profit companies, with the remaining 199 being authored by funding/advocacy agencies (95, 44%), WHO (includes partnerships with UNICEF and the Global Antibiotic Research and Development Partnership; 51, 24%), PDPs (34, 16%), and public institutes and universities (17, 8%) (Fig. 2). The top five organizations that reported product profiles are shown below (Table 3).

**Table 3:** The top five organizations reporting product profiles in the Health Product Profile Directory (May 2019)

Global Health Innovative Technology Fund	62 profiles	Diagnostics (18), Drug (26), Vaccine (18)
World Health Organization (incl. Partnerships with UNICEF and Global Antibiotic Research and Development Partnership)	51 profiles	Diagnostics (17), Drug (3) Vaccine (21), Digital Health (7), Drug Regimen (3)
Bill and Melinda Gates Foundation	24 profiles	Drugs (4), Vaccine (13) Drug Regimen (1), Injectable/implant (3), Not Defined (3)
Serum Institute	12 profiles	Vaccines (12)
Drugs for Neglected Diseases Initiative	10 profiles	Drugs (8), Vaccine (2)

The distribution of profiles is heavily weighted to those agencies in the not-for-profit sector. This is to be expected and reflects the inclusion criteria for the HPPD, with its focus on global health where markets are limited. Additionally, there might be a greater reluctance to share specific product profile information in a TPP by a commercial entity if that would compromise intellectual property protection. However, now the HPPD is launched, this concern may reduce when users can assess for themselves that the summary information can be presented in a way that would highlight what is in a pipeline but not require them to reveal commercially sensitive information.

**Fig. 3:**
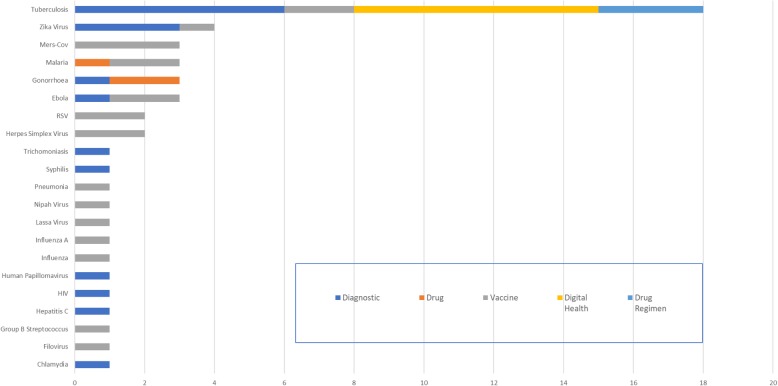
Product profiles authored by WHO, includes partnerships with UNICEF, Global Antibiotic Research and Development Partnership (May 2019, *n* = 215)

Finally, an analysis of those profiles authored by WHO are presented (Fig. 3). This demonstrates the potential of the HPPD to undertake a high-level analysis of the portfolio for an organisation. For WHO, the analysis presented here represents all the known profiles that have been published at the time of publication. The disease profile reflects the global analysis with a strong emphasis on infectious diseases and no profiles in the NCD area.

## Discussion

It is important to note that the profiles reported here are those that are available in the public domain or have been disclosed by a contact at the authoring institution. There are many more products in the pipeline for which product profiles, and more specifically TPPs, might exist. An analysis of the product pipeline for HIV, TB, malaria and the neglected tropical diseases undertaken in 2017 reports a total of 685 product candidates [12]. Therefore, the content of the HPPD, which has 210 profiles covering the same diseases, suggests that coverage might be as low as 31% of what could be available. However, as we have noted, all the profiles here could be considered strategic documents and do not provide references that relate to specific products in the pipeline. Therefore, this low coverage could be the result of comparing two different phases of the innovation cycle with the HPPD providing upstream guidance and product pipeline analysis measuring actual R&D activity. Moving forwards, we would want to be able to track the impact of a strategic product profile on R&D activity, particularly those profiles authored by WHO, to assess if they are meeting their stated aim of stimulating innovation in areas of public health need. For example, the WHO R&D Blueprint for emerging pandemic diseases has led to the creation of detailed product profiles in diagnostics and vaccines. The creation of these profiles, with a relatively standardized format, have been adopted as the research priorities for the Coalition for Epidemic Preparedness Innovations (CEPI), which has subsequently provided $350 million in R&D funding [13]. Thus, in time, it should be possible to trace the degree to which these strategic profiles have stimulated and shaped R&D funding. For the neglected tropical diseases with less coverage in the HPPD, the indication is that more work is needed to create a similar set of precise health product profiles to better communicate product R&D priorities in this area.

For NCDs, medical devices and reproductive health, the number of profiles in the HPPD is below 2%. Thus, if product profiles are to become the norm for describing product R&D priorities, then much more development in needed in these disease areas.

A key consideration for inclusion of a health product profile in the HPPD is that it describes a product for which there is a limited market to provide the incentive for traditional R&D. This means that access and affordability should be embedded within a product profile and particularly in any prospective profile describing Preferred Product Characteristics. In this sample, we were able to observe some of the elements that relate to access, such as target population, indication and intended use in a low-resource setting but stating a specific price point for the final product is absent from most product profiles in this sample. Ten diagnostic profiles authored by WHO include information on a minimal and optimal target price per test, these target price points range from $1–5 (excluding the cost of a device or reader). Whilst recognising the complexities, stating a maximum price for a product to ensure affordability in the target countries should be an important consideration in all product profiles aimed at use in low- and middle-income countries. For example, the Meningitis Vaccine Project identified affordability as a key guiding principle when it developed the product development plan for a new meningococcal vaccine in 2002. Following input from public health leaders in the African countries where the vaccine was needed, the target for the maximum cost per dose for the new vaccine was set at no more than $0.50. Setting this price target early is cited as one of the major contributing factors that subsequently guided the research, development, uptake and use of this vaccine (MenAfriVac) [14].

The variation of formats and detail in the product profiles and the widespread use of TPP to describe the types of product profile with varying objectives means that, without the standardisation provided by HPPD, it would not be possible for any analysis or comparison to take place. The HPPD provides that opportunity for the first time in an online resource. While broad analysis can reveal significant gaps, for example, the lack of coverage of the NCDs, more work needs to be done to develop standards if the use of product profiles is to become the norm in describing priorities in global health product R&D.

If product profiles are to drive innovation in the future, then an ideal scenario can be imagined. Priorities for global health R&D would be identified following an evaluation of products in the current health system. By incorporating input from a wide range of stakeholders, including end users and patients, a consensus document or roadmap would set out the value proposition. The importance of gaining input from end-users is again highlighted by the Meningitis Vaccine Project as it was the feedback from the officials in the target Africa countries that made it clear that, unless the final vaccine cost less than $0.50 a dose, they could not afford to use it [14]. This roadmap would describe the public health need and how a product of a certain type would best address that need.

**Fig. 4:**
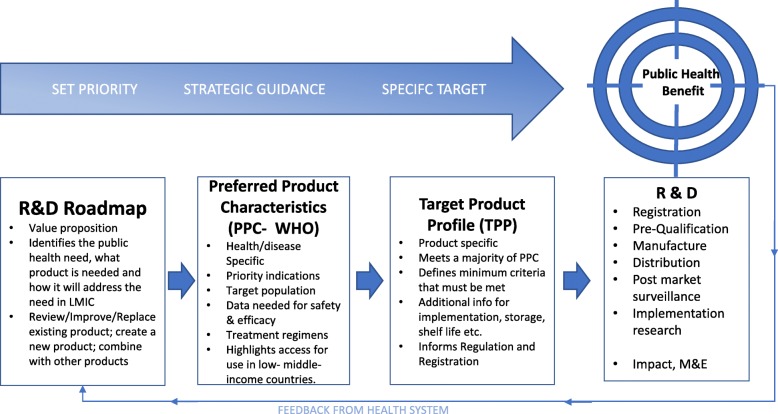
The role of health product profiles in shaping research and development (adapted from [15])

The technical specification describing these preferred characteristics would be published in a PPC document. In global health, a WHO-PPC should represent the highest level of global consensus. The PPC would describe an ideal and set a measure or benchmark against which new products could be assessed. Following publication of a PPC, we would want to see this stimulate innovation as evidenced by several TPP candidates entering the pipeline (Fig. 4) [15].

## Conclusion

The HPPD clearly demonstrates its potential to meet many of the aims it was designed for. It brings together health profiles (documents that describe PPCs and TPPs) in a searchable database. The profiles are written to describe priority health products (drugs, diagnostics, vaccines and other devices) that are essential to tackle pressing health needs with a focus on populations in low- and middle-income countries. Currently, these documents are found in varied formats scattered across webpages, in repositories or published in journals. Standardising the documents to a template and including them in a free-to-access directory has enabled comparison and analysis across the profiles and the disease area they target. As awareness of the HPPD grows, this should increase access to the profiles and therefore their impact. It is important to note the HPPD only published a summary of the original document. If a reader wishes to understand in more detail how a profile was developed and, for example, who was consulted during that development, then they should always read the original document.

The information contained in the HPPD should inform any discussion of R&D priorities in global health, particularly the need to include considerations of access, such as price, at an early stage in the innovation cycle. All the current profiles authored by WHO are indexed and clearly labelled, which will enable all interested stakeholders to identify and access these documents. These documents aim to provide a global consensus to guide and coordinate global health R&D efforts, particularly where there is market failure. The HPPD therefore addresses what has been termed a public health failure by industry, i.e. the absence of clear, technical documents describing, in specific details, what characteristics a new health product should have if it is to be effective in tackling a disease in low-resource settings.

The HPPD is easy to navigate and this enables a user to identify where a profile exists and, in most cases, find more detailed information on that product profile. The resource is open access, so all the data can be readily downloaded into Excel for further analysis.

The analysis presented here highlights the need to develop and agree standards in the production of health product profiles, particularly if the use of strategic profiles is to become a norm for WHO-authored PPCs, with their objective to describe global health R&D priorities.

In particular, it is recommended that all PPC profiles should include statements that define the value proposition more precisely. Whilst it may prove difficult to define exact price points that apply for all products in all countries and for all populations/subpopulations, more work needs to done to define what ‘affordability’ means. All profiles should also include a statement regarding safety. The authors are aware that WHO is developing guidance for the development of WHO-authored profiles. These issues could be further addressed by a moderation process requiring these fields to be completed as a mandatory part of submission to the HPPD.

The utility of the HPPD will grow as more content is ingested and this improvement in transparency should enable us to track resource flows to those identified priorities. In time, this should lead to a greater coordination of the resources we have available to tackle pressing global public health needs.

Combining the analysis from HPPD to a mapping of funds available for R&D, for example, as measured by the annual G-FINDER survey for neglected diseases, and products in the R&D pipeline as published by the WHO Global Observatory on Health R&D, would create a better overview of global health priorities and how they are supported [16, 17]. This is the subject of ongoing work by TDR with its partners Duke University and Policy Cures Research due for publication later in 2019. Such analysis and increased transparency should take us a step closer to measuring and improving coordination of efforts in global health R&D.

## Supplementary information

**Additional file 1.** Download of data contained in the Health Product Profile Directory, 5 May 2019.

## Data Availability

The Health Product Profile Directory (HPPD) https://www.who.int/tdr/product-profile-directory is published as open access under the Creative Commons Attribution-NonCommercial-ShareAlike 3.0 IGOlicence (CC BY-NCSA 3.0 IGO; https://creativecommons.org/licenses/bync-sa/3.0/igo). All data analysed in this paper is available directly from the HPPD and is also provided in Additional file 1.

## Abbreviations

HPPD: Health Product Profile Directory
NCDs: non-communicable diseases
PDP: Product Development Partnership
PPC: Preferred Product Characteristics – used by WHO
R&D: research and development
TB: tuberculosis
TDR: The Special Programme for Research and Training in Tropical Diseases
TPP: Target Product Profile
